# Role of the Extracellular Matrix in Alzheimer’s Disease

**DOI:** 10.3389/fnagi.2021.707466

**Published:** 2021-08-27

**Authors:** Yahan Sun, Sen Xu, Ming Jiang, Xia Liu, Liang Yang, Zhantao Bai, Qinghu Yang

**Affiliations:** College of Life Sciences and Research Center for Resource Peptide Drugs, Shaanxi Engineering and Technological Research Center for Conversation and Utilization of Regional Biological Resources, Yanan University, Yanan, China

**Keywords:** Alzheimer’s disease, extracellular matrix, synaptic transmission, amyloid-β plaque, tau-protein, oxidative stress, inflammation

## Abstract

Alzheimer’s disease (AD) is a neurodegenerative disease with complex pathological characteristics, whose etiology and pathogenesis are still unclear. Over the past few decades, the role of the extracellular matrix (ECM) has gained importance in neurodegenerative disease. In this review, we describe the role of the ECM in AD, focusing on the aspects of synaptic transmission, amyloid-β-plaque generation and degradation, Tau-protein production, oxidative-stress response, and inflammatory response. The function of ECM in the pathological process of AD will inform future research on the etiology and pathogenesis of AD.

## Introduction

Alzheimer’s disease (AD) is the most common cause of dementia and the fourth leading cause of death after cardiovascular disease, cancer, and acquired immune deficiency syndrome (AIDS). AD symptoms usually manifest as memory loss, cognitive decline, personality changes, and other neurological indications including depression, restlessness, anxiety, and aggressive behaviors. The typical pathological characteristics of AD are amyloid-beta (Aβ) deposits, neurofibrillary tangles (NFTs), synapse and neuron loss, glial activation, and disorganization of the extracellular matrix (ECM). Numerous hypotheses, such as the Aβ hypothesis (Hardy and Selkoe, 2002), the oxidative stress hypothesis (Maccioni et al., 2010), and the Tau protein abnormal phosphorylation hypothesis (Terry and Buccafusco, 2003), have attempted to explain the etiology of AD and have targeted both genetic and environmental factors (Wenk, 2003), yet the causative mechanisms of this disease remain elusive.

The ECM is a structural network composed of various macromolecules secreted by cells, namely collagen, elastin, fibronectin, laminin, glycoproteins like tenascin-R (TNR), and tenascin-C (TNC), glycosaminoglycans (GAGs), and proteoglycans (Frantz et al., 2010). In the central nervous system (CNS), neural ECM molecules produced and released by neurons and surrounding cells (astrocytes, oligodendrocytes, etc.) are extensively accumulated in the extracellular space (Testa et al., 2019). Proteoglycans consist of a core protein covalently attached to one of five GAG chains (Yanagishita, 1993), namely chondroitin sulfate, keratan sulfate, dermatan sulfate, heparan sulfate, and the non-sulfated GAG hyaluronan. The carboxyl group of uronic acid on the surface of hyaluronic acid (HA) has a large amount of negative charge, and its repulsive effect causes the whole molecule to stretch and swell; the hydrophilic group can also combine with a large number of water molecules to make the matrix isotonic and edema.

These form a viscous colloid and generate swelling pressure, giving the tissue good elasticity and resistance to pressure, as well as interfere indirectly with parameters of neuronal excitability by means of their influence on extracellular space volume (Arranz et al., 2014). Chondroitin sulfate proteoglycan (CSPG) usually acts as a barrier molecule and plays an important role in embryonic development and plasticity of the central nervous system in adulthood (Miyata and Kitagawa, 2016). Astrocytes promote the growth and development of the brain by releasing factors such as TNC (Pollen et al., 2015), which regulates neuronal development. TNR is a membrane-bound connexin that is closely related to the formation and stability of the perineural network (Suttkus et al., 2014). The ECM serves to maintain cell morphology and structure (Blumenthal et al., 2014) and is involved in the survival, differentiation, development, and migration of nerve cells. There are three main types of ECM in the brain and spinal cord: the ubiquitously present “loose” ECM; cell membrane-bound molecules such as TNR; and perineuronal nets (PNNs) that wrap around specific neurons (Celio et al., 1998; Deepa et al., 2006; Soleman et al., 2013). As a critical component of the extracellular neural space, the ECM is closely related to several neurodegenerative diseases, especially AD. Many studies have reported alterations in the expression profile of ECM proteins in early-onset AD. Table 1 lists changes in ECM protein expression during the development of AD. Such changes before the occurrence of AD could disturb the homeostasis of the nervous system and promote the onset of AD.

**Table 1:** Expression of extracellular matrix (ECM) component changes in the pathological state of AD.

ECM component	Expression in AD	References
Hyaluronic Acid (HA)	↑	Nielsen et al. (2012), Nägga et al. (2014), Li et al. (2017), and Reed et al. (2019)
Heparin Sulfate Proteoglycan (HSPG)	↑	Van Horssen et al. (2003), Shimizu et al. (2009), and Lorente-Gea et al. (2020)
Chondroitin Sulfate Proteoglycan (CSPG)	↑	Shimizu et al. (2009) and Goetzl et al. (2019)
Keratan Sulfate Proteoglycan (KSPG)	↓	Lindahl et al. (1996) and Snow et al. (1996)
Dermatan Sulfate Proteoglycan (DSPG)	↑	Shimizu et al. (2009) and Genedani et al. (2010)
Tenascin C (TNC)	↑	Xie et al. (2013), Hondius et al. (2016), and Hasanzadeh et al. (2021)
Tenascin R (TNR)	↑	Manavalan et al. (2013) and Végh et al. (2014)
Reelin	↓	Herring et al. (2012), Mota et al. (2014), and Shabani et al. (2018)

*The expression of KSPG and reelin decrease, whereas that of HA, HSPG, CSPG, DSPG, TNC, and TNR increase*.

**Figure 1:**
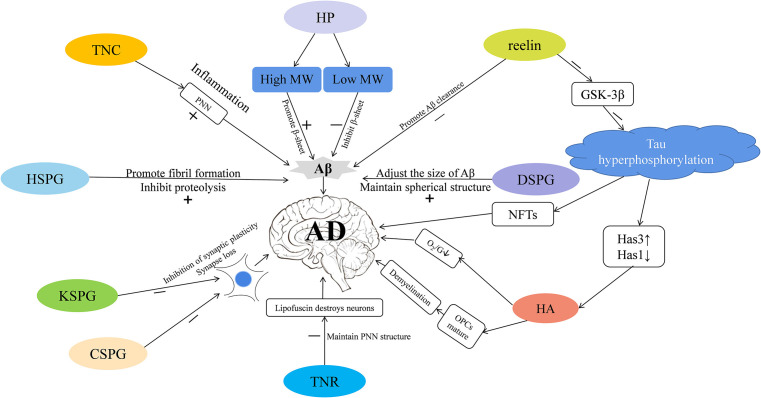
The extracellular matrix (ECM) participates in the progression of Alzheimer’s disease (AD) *via* different mechanisms. Tenascin-C (TNC) is increased in AD—it increases the stability of perineuronal nets (PNNs) to reduce the clearance of amyloid-β (Aβ), and participates in inflammatory pathways that leads to the occurrence of AD. PNNs also wrap around neurons and protect them from the neurotoxic effects of Aβ. Heparin (HP) is increased in AD—high molecular-weight (MW) HP promotes the formation of β-sheet secondary structure, and low molecular-weight (MW) HP inhibits it, which in turn affects the production of Aβ. Heparin sulfate proteoglycans (HSPGs) are increased in AD; they promote the formation of Aβ fibrils, inhibit amyloid hydrolysis, and promote the production of Aβ. Dermatan sulfate proteoglycans (DSPGs) are increased in AD—they may modulate the size of Aβ plaques, maintain the spherical structure of Aβ, thereby regulating the occurrence of AD. Reelin is decreased in AD—it can promote the clearance of Aβ, and inhibit the expression of GSK-3β that phosphorylates the Tau protein and promotes neurofibrillary tangle (NFT) formation, thus mediating the occurrence of AD. Tau hyperphosphorylation reduces hyaluronan synthase 1 (Has1), increases hyaluronan synthase 3 (Has3), and up-regulates short-chain Hyaluronic acid (HA). HA is increased in AD; it inhibits the maturation of OPCs, causes demyelination, reduces the supply of brain oxygen and glucose (O2/G), and may promote the occurrence of AD. TENASCIN-R (TNR) is increased in AD; it increases PNNs stability and may prevent lipofuscin from destroying neurons. Keratan sulfate proteoglycan (KSPG) is decreased in AD and chondroitin sulfate proteoglycan (CSPG) is increased; both these macromolecules may inhibit synaptic plasticity, cause synapse loss, and promote pathological damage in AD.

As we communicate in this review, different types of ECM may participate in the pathology of AD *via* different pathways (Figure 1). Here, we focus on the role of the glycosaminoglycans and proteoglycans in the occurrence of AD from the aspects of synaptic signal transmission, Aβ plaque generation and degradation, Tau protein production, oxidative stress response, and the inflammatory response. Taken together, these pieces of evidence strongly support that ECM dysregulation is closely associated with AD, and future research can better inform efforts to study the etiology and pathogenesis of AD.

## The ECM Inhibits Synaptic Transmission and Aggravates the Pathological Process of AD

Synaptic changes are a frequent occurrence in neurodegenerative diseases and are likewise observed in the early stages of AD. The loss of synapses in the AD brain is closely associated with cognitive dysfunction and the decline of learning and memory. Many dendritic spine abnormalities and a decrease in synapse number have been observed in cognition-related brain areas, like the prefrontal cortex and hippocampus, in the early stages of AD (Vertes et al., 1999).

AD is also called a “synaptic degeneration disease” (Selkoe, 2002). The critical balance between excitatory and inhibitory neurotransmission, which is key to normal cognitive function, is completely disrupted in the AD state (Boyce et al., 2016). Some reports implicate keratan sulfate proteoglycan (KSPG) in the regulation of synaptic function (Snow et al., 1996). The level of KSPGs in the cerebral cortices of AD patients is much less than in healthy individuals (Table 1, Lindahl et al., 1996). KSPGs have been reported as primarily located at synapses and dystrophic neurites within neuritic plaques of AD and the normal, aged brain (Snow et al., 1996). Interestingly, the core protein of KSPG, SV2Proteoglycan (SV2PG), is mainly located on the synaptic vesicle membrane (Buckley and Kelly, 1985), indicating that this position may be a potential site where the keratan sulfate chain attaches to carry out the functions of the KSPG (Scranton et al., 1993). In addition to being present in most synapses, the SV2PG amino-acid sequence has homology with other proteins identified as transporters (Bajjalieh et al., 1992; Feany et al., 1992; Gingrich et al., 1992), suggesting a potential function in synaptic vesicle transport. The localization of SV2PG on dystrophic neurites indicates that changes in neurotransmission may mainly involve abnormal neurites in AD (Buckley and Kelly, 1985; Snow et al., 1996). The lack of highly sulfated KSPGs in AD may affect neurotransmission and weaken communication between neurons, thereby impairing the learning and memory of AD patients (Figure 1; Lindahl et al., 1996).

**Figure 2:**
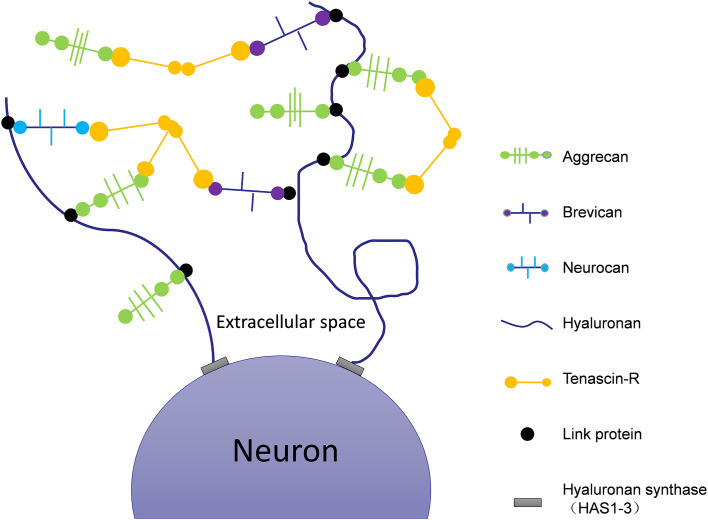
Schematic of the molecular composition of PNNs. Hyaluronan synthases are located at the neuronal cell membrane, synthesizing hyaluronan and secreting it into the perineuronal area. Members of the lectican family (aggrecan, neurocan, and brevican) bind to the hyaluronic backbone. Link proteins stabilize the binding of CSPGs to hyaluronan. Tenascin-R (TNR) crosslinks the lecticans to form stable PNNs.

In addition to neurotransmission, synapse number and neuronal plasticity are also affected in AD (Vertes et al., 1999). Hyaluronic acid (HA) is one of the main components of the ECM (De La Motte and Drazba, 2011) that is linked to AD pathology *via* its effects on neuronal function and plasticity. The expression of HA increases with the progression of AD (Table 1, Reed et al., 2019). HA can inhibit the maturation of oligodendrocyte progenitors (Back et al., 2005). Abnormal expression of HA will demyelinate neurons, impair the transmission of nerve signals, limit remyelination, and cause white matter lesions (Figure 1; Montine et al., 2012). In human magnetic resonance imaging studies (Back et al., 2011), it was observed that white matter lesions in the medial prefrontal cortex (mPFC) were significantly associated with vascular injury and co-localized with areas rich in HA. The atypical increase of HA content in the brains of AD patients may cause vascular injury, a decrease of cerebral blood flow, decrease of oxygen and glucose supply to the brain, synapse loss, decline of neuronal function, and ultimately aggravate the cognitive deficits of AD patients (Figure 1; Park et al., 2014). Finally, HA is also present in the core of PNNs (Figure 2), which wrap around neurons and inhibit neuronal plasticity during aging and disease (Sorg et al., 2016).

It was reported that Aβ plaques produced in the brains of AD patients can cause changes in synaptic plasticity (Abramov et al., 2009). According to these reports, the chondroitin sulfate proteoglycan (CSPG) content in the brains of AD patients is higher than that of healthy individuals (Table 1, Goetzl et al., 2019). Chondroitin sulfate 4 (C-4) is expressed in the core of senile plaques (SPs) and neurofibrillary tangles (NTFs), non-chondroitin sulfate (C-0) is expressed in intracellular NTFs and dystrophic neurites of SPs, and chondroitin sulfate 6 (C-6) is expressed in the core of NTFs and around SPs (Dewitt et al., 1993). The combination of CSPGs and the inhibitory receptor protein tyrosine phosphatase σ (PTPσ) inhibits neuronal plasticity (Paveliev et al., 2016). Injecting chondroitinase ABC (ChABC) into the hippocampus of AD mice cuts off the CSPG network, thereby reducing Aβ plaques in the molecular layer, restoring synaptic density around Aβ plaques, enhancing synaptic plasticity, and finally improving the long-term memory capabilities of AD mice (Figure 1; Howell et al., 2015). Interestingly, the injection of ChABC into the secondary visual cortex (V2L) to relieve CSPG load can reduce long-term spatial memory in rats without affecting short-term memory, suggesting that the mechanisms of long-term and short-term memory are distinct. Moreover, rats’ recall of remote, but not recent, visual fear memories is dependent on intact PNNs in V2L (Thompson et al., 2018).

The PNNs are highly stable structures relieved from constant renewal as they are not exposed to the catabolic intracellular environment. Therefore, it has been proposed that PNN may continue to be a material framework for stabilizing long-term memory (Tsien, 2013). Moreover, the PNNs promote the fast-spiking activity of Parvalbumin-positive (PV+) interneurons, and consequently the excitatory–inhibitory balance of neural networks required for cognitive functions (Lensjø et al., 2017), which is essential for consolidation and retrieval of memories (Xia et al., 2017). The efficacy of ChABC in AD model and non-AD model animals may be caused by differences in CSPG content (Goetzl et al., 2019). In non-AD model animals, CSPG expression is relatively normal and sufficient to maintain the balance of the PNN network. Using ChABC to break up CSPG affects the stability of PNN and the material framework of long-term memory (Tsien, 2013), resulting in a decrease in long-term memory. Indeed, in AD model animals, the content of CSPG increases (Goetzl et al., 2019), and the normal structure of PNN can be restored by injection of ChABC so as to restore long-term memory.

In the brains of AD animal models, PNNs surround Aβ plaques, blocking the degradation of amyloid and inhibiting the growth of neurites (Hockfield et al., 1990; Pizzorusso et al., 2002; Mcrae et al., 2007). This restricts synaptic plasticity and affects the transfer of information between neurons. Tenascin-R (TNR), which is involved in the formation of PNNs (Figure 2), is increased in the brains of AD patients (Végh et al., 2014). The main components of PNNs are lecticans (aggrecan, brevican, neurocan, and versican), phosphacan, hyaluronan, TNR, and link proteins (cartilage link protein Crtl-1/HAPLN-1 and brain link protein Bral2/HAPLN-4; Figure 2). They interact with each other to provide the molecular backbone of the PNN, thereby creating a stable scaffold around the cell bodies and proximal dendrites of neurons (Figure 1; Suttkus et al., 2014). PNNs inhibit the outgrowth of neurites (Hockfield et al., 1990; Pizzorusso et al., 2002; Mcrae et al., 2007), which may lead to restricted synaptic plasticity and defective neurotransmission over the course of AD, aggravating the disease’s pathological damage.

## The ECM Is Involved in the Formation and Degradation of Aβ Plaques

When Aβ oligomers form, they become anchored to the cell membrane and result in the breakdown of the phospholipid bilayer (Friedman et al., 2009). They also interact with the hydrophobic region of the cell membrane to form transmembrane pores, which leads to the outflow of cell contents and an imbalance of ion homeostasis (Österlund et al., 2019). High molecular-weight heparin (HP) can bind to Aβ and promote the conversion of Aβ peptides from random coils to β sheets (Bruinsma et al., 2010), thereby promoting amyloid aggregation and fibrosis and stabilizing the formed senile plaques. On the contrary, low molecular-weight HP can reverse the process of amyloidosis by hindering the formation of β sheets (Walzer et al., 2002), and thus prevent the neuropathic process induced by Aβ (Figure 1). This fact indicates that HP proteins of different molecular weights play different roles in the course of AD. Studies have shown that removing the O-sulfuric acid group in HP weakens its role in promoting Aβ aggregation. Moreover, when the sulfuric acid group is completely removed, its promotion of Aβ aggregation disappears completely (Ariga et al., 2010), indicating that the sulfuric acid group is very important for the aggregation of Aβ.

The ECM-component HSPG is also increased in the brains of AD patients (Table 1, Van Horssen et al., 2003). Agrin is an HSPG containing nine protease-inhibiting regions (Stone and Nikolics, 1995; Castillo et al., 1999) and prevents proteases from degrading the Aβ protein. Agrin can enhance the formation of Aβ fibrils *in vitro* and can protect fibrils against proteolysis (Gupta-Bansal et al., 1995). As AD progresses, immature, non-fibrillar Aβ plaques eventually develop into mature, fibrillar Aβ plaques (Figure 1; Selkoe, 1991). This finding supports the contribution of HSPG to the formation of mature Aβ fibrils in AD. The pathogenic mechanism of this process operates as follows: Agrin prevents proteases from degrading the Aβ protein, the continuous deposition of which leads to the disease. Studies have shown that the degree of sulfation of the HSPG is essential to promote the formation of fibrils of Aβ (Cohen et al., 1997) or other amyloid peptides (Castillo et al., 1998). The sulfation pattern of HSPG does not occur randomly (Dennissen et al., 2002). Five anti-HS antibodies obtained through phage-display technology were used to label the topological structure of HSPG epitopes, and it was found that each antibody recognized a unique epitope structure of sulfate groups. This shows that the position of the sulfate group is very important for the function of the HSPG (Ten Dam et al., 2003, 2006; Kurup et al., 2007; Wijnhoven et al., 2008). Highly N-sulfated and O-sulfated HS may participate in the initiation of Aβ aggregation (Hileman et al., 1998) or stabilize previously formed Aβ (Holm Nielsen et al., 2000). The His-His-Gln-Lys amino-acid sequence on the Aβ peptide chain is considered the binding site for the interaction between glycosaminoglycans and Aβ, among which His13 may be the main site for the function of the sulfate group.

There are different structures of Aβ aggregates (namely, monomers, oligomers, and fibers) in the AD brain, indicating that there may be a connection between the progression of AD and the structure of Aβ (Gremer et al., 2017). Therefore, understanding what maintains the spherical structure of Aβ aggregates will provide a new perspective for the treatment of AD. Dermatan sulfate proteoglycans (DSPGs) are upregulated in the brains of AD rats (Table 1, Genedani et al., 2010). Using a variety of antibodies to identify decorin, the core protein of DSPG, in the brains of AD patients, positive staining was found in the filamentous structure of amyloid deposits and NFTs. Unlike HSPG, which tends to be evenly distributed in neuritic plaques (NPs) containing amyloid, decorin is mainly distributed around spherical amyloid plaques and the edges of amyloid fiber bundles. The lack of decorin in the center of certain amyloid plaques indicates that the distribution of DSPGs is spatially restricted (Snow et al., 1992). The abnormal distribution of decorin around amyloid plaques suggests that DSPGs may help regulate the size of the amyloid plaques in NPs or maintain its spherical structure (Figure 1; Obrink, 1973; Flint et al., 1984; Vogel et al., 1984; Scott, 1988). Moreover, the close relationship between blood vessels and amyloid plaques (Nortley et al., 2019) suggests that vascular-wall proteoglycans may be associated with Aβ deposits or NFTs in nearby plaques. Different DSPGs were also shown to have different binding affinities for amyloid (Buée et al., 1993). Taken together, this evidence supports that DSPGs play an important role in the formation of amyloid plaques and the pathogenesis of AD. After the induction of β-amyloid fibrils in the rat striatum, the DSPG content and the total charge density were shown to increase significantly (Genedani et al., 2010), indicating that the production of Aβ in the AD brain may trigger a responsive change in DSPG expression.

It has been reported that the expression of reelin and its glycosylation pattern is altered in the cerebrospinal fluid of AD patients (Botella-López et al., 2006). Specifically, there is evidence to show that the depletion of reelin is an early event in AD pathology (Table 1). A recent study showed that reelin and its downstream signaling members APOER2, VLDLR, and DAB1 are all affected in AD. Since reelin depletion occurs before the onset of Aβ pathology, the decline of reelin may play a role in the pathological precipitation of Aβ (Herring et al., 2012). Consistent with the above, the expression of reelin in the entorhinal cortex of transgenic mice and humans with AD is reduced (Chin et al., 2007). Further, reelin has been shown to inhibit Aβ production, promote Aβ clearance, and prevent Tau protein hyperphosphorylation (Figure 1; Dulabon et al., 2000; Brich et al., 2003; Deane et al., 2008).

## Interaction Between ECM and Tau Protein

Tau belongs to the microtubule-associated protein (MAP) family, which can effectively stabilize microtubules and promote retrograde (periphery to the nucleus) and anterograde (nucleus to outer periphery) axonal transport (Kadavath et al., 2015; Wang and Mandelkow, 2016). Therefore, the normal function of tau is essential for neuronal transport and synaptic structure (Avila et al., 2016; Guo et al., 2017). Tau hyperphosphorylation can lead to the deterioration of the dendritic structure and axonal transport, as well as the depolymerization of microtubules (Baudier and Cole, 1987).

HA synthases (HAS), including Has1, Has2, and Has3, are widely expressed in the central nervous system of mice. All types of HAS are located in the cell bodies of neurons, but only Has1 is found in axons. In TauP301S transgenic mice where tau protein is overexpressed, it was found that Has1 is no longer localized to axons, similar to the redistribution of Has1 expression observed in the brains of AD patients. Additionally, in the brains of TauP301S transgenic mice, Has1 expression decreases, whereas Has3 expression increases, leading to the upregulation of short-chain HA in the ECM (Figure 1; Li et al., 2017). The localization of Has1 depends on intact microtubules, and its mislocalization caused by the hyperphosphorylation of Tau disrupts the balance of ECM components, promotes ECM recombination, and inhibits the formation of PNNs. Under such conditions, neurons become more susceptible to the invasion of neurotoxic substances such as Aβ, which inhibit synaptic remodeling and aggravate AD.

Reelin is activated through the SFK/PI3K/Akt pathway to inhibit the expression of GSK-3β and prevent Tau phosphorylation, another pathological occurrence in AD. Recent studies have found that GSK-3β is the most effective Tau protein kinase and can promote an abnormal increase in Tau phosphorylation (Figure 1; Ohkubo et al., 2003). Consistently, studies have shown that the expression of GSK-3β colocalizes with NFTs, and both its expression and activity are significantly increased in the brains of AD patients.

## The ECM Resists Oxidative Stress and Reduces AD Damage

Under healthy physiological conditions, the reactive oxygen species (ROS) metabolized by the body is maintained in a steady state of redox by the body’s natural antioxidant system. The hypothesis of free radical damage in AD postulates that (Cabungcal et al., 2013) the body’s defense systems are weakened with disease progression, causing the accumulation of a pathologically high level of free radicals, which react with unsaturated fatty acids in the cell membrane to form lipid peroxides such as malondialdehyde. Malondialdehyde combines with proteins, nucleic acids, and other biological macromolecules to form insoluble lipofuscin deposits in cells (Kun et al., 2018). Lipofuscin can damage cell structure, disrupt cell metabolism, accelerate cell senescence and death, as well as affect learning and memory (Giaccone et al., 2011; Kwon et al., 2016). As shown in Table 1, as the course of AD progresses, the expression of tenascin-R increases (Végh et al., 2014). Neurons in the cortex and subcortex are surrounded by PNNs, which protects them from neurofibrillary degeneration (Brückner et al., 1999; Morawski et al., 2012). Studies have shown that compared with unprotected neurons, those surrounded by PNNs are less affected by the accumulation of lipofuscin present in AD (Morawski et al., 2004). Since lipofuscin is an indicator of oxidative stress and aging (Sohal and Brunk, 1989), this finding indicates that the PNN may have a protective function against oxidative stress-induced neurodegeneration in AD.

Aβ-induced cerebrovascular (CV) deficits are mediated by ROS (Iadecola et al., 2009). Application of exogenous, soluble Aβ (Aβ1–40 and Aβ1–42 monomers) onto isolated mouse cerebral arterioles leads to significant oxidative stress and vasomotor dysfunction, and anti-ROS strategies markedly improve these CV deficits (Dietrich et al., 2010). Tg2576 mice in age from 2–3 months with elevated levels of endogenous, soluble Aβ species display substantial oxidative stress and CV deficits (Park et al., 2005). HSPGs are an attractive upstream candidate for Aβ-induced ROS production and CV dysfunction in AD. HSPGs bind Aβ with high affinity and promote their intracellular uptake in multiple cell types (Sandwall et al., 2010), including human cerebral vascular smooth muscle cells (VSMC; Kanekiyo and Bu, 2009). Therefore, HSPGs could be key mediators of Aβ1–42-induced oxidative stress and Aβ1–40-induced VSMC dysfunction. Aβ could interact with the cell surface or extracellular-matrix HSPGs, leading to intracellular calcium influx and ROS production. Toxic ROS species could directly damage the VSMC contractile machinery, leading to a hypercontractile phenotype that would reduce the supply of oxygen and glucose to the brain, thereby aggravating the pathology of AD (Reynolds et al., 2016).

Chondroitin sulfate (CS) oligosaccharides have also been tested as a therapeutic strategy in AD. They block Aβ-induced oxidative stress in SH-SY5Y cells and mitochondrial dysfunction (Zhao et al., 2020) in AD mice. They also inhibit oxidative stress, production of pro-inflammatory cytokines, and activation of the toll-like receptor pathway in Aβ-injured BV2 microglia (Zhao et al., 2020). CS oligosaccharides were reported to significantly suppress Aβ-induced oxidative stress by increasing the activity of antioxidant enzymes, including SOD and GSH-Px. It has been suggested that CS oligosaccharides may bind Aβ fibrils and inhibit them from interacting with cell and mitochondrial membranes; this process may be associated with the molecular weight of the CS oligosaccharides (Zhang et al., 2018). As such, modulation of ROS and identification of the upstream inducers of Aβ-mediated ROS production will be instrumental in designing novel therapies to prevent Aβ-induced CV dysfunction and to ameliorate the effects that these vascular deficits have on AD dementia.

## The ECM Participates in the Inflammatory Response and Regulates Inflammatory Damage in AD

Neuroinflammation is a result of the biological response of microglia and invading immune cells to harmful substances (Gendelman, 2002). Studies have shown that the pathology of AD (including Aβ accumulation and tau hyperphosphorylation) can cause inflammation in susceptible areas (Akiyama et al., 2000; Hamelin et al., 2016). The low molecular-weight HA fragments synthesized by Has3 or degraded by hyaluronidase can cause inflammation (Simpson et al., 2015). In severe AD, tumor necrosis factor (TNF)-stimulated gene-6 (TSG-6) was shown to be significantly increased and was found in NeuN-positive neurons and microglia (Hanger et al., 2014). TSG-6 is believed to reduce inflammation and provide tissue protection for various organs (Day and Milner, 2019). It is expressed in the brains of adult rodents and may become cross-linked with HA during the formation of glial scars in the central nervous system (Coulson-Thomas et al., 2016), thereby changing the structure of HA (Baranova et al., 2011) to one that enhances interaction with its receptors (Lesley et al., 2004; Lawrance et al., 2016; Richter et al., 2018). In this way, TSG-6 may help HA to alleviate neuroinflammation during AD (Day and Milner, 2019).

In AD, it has been reported that microglia (Krstic and Knuesel, 2013; Niraula et al., 2017) and astrocytes (Batarseh et al., 2016) can enhance the phagocytosis of harmful substances to resist the damage caused by AD; however, other studies suggest they secrete pro-inflammatory cytokines, which can damage brain cells and aggravate the process of AD. Studies have shown that a tenascin-C (TNC) deficiency reduces pro- but enhances anti-inflammatory activation in the mutated APP-transgenic mouse brain, associated with a reduced cerebral Aβ load and higher levels of postsynaptic density protein 95 (PSD-95; Xie et al., 2013). In other words, TNC transforms the neuroinflammatory process from pro- to anti-inflammatory (Xie et al., 2013). Reducing the level of TNC in the brains of AD mice significantly increased the number of microglia and macrophages, and decreased the activity of β-secretase and γ-secretase in the hippocampus and cortex (Heneka et al., 2015), which can effectively reduce the course of synapse damage in AD. This finding indicates that the pathogenesis of AD is not limited to neurons, but also includes interactions with the immune mechanisms of the brain. Misfolded and aggregated proteins bind to pattern-recognition receptors on microglia and astrocytes, which trigger the innate immune response characterized by the release of inflammatory mediators, thereby aggravating the progression of AD (Heneka et al., 2015). As an endogenous activator, TNC can accumulate in the AD brain and cause chronic inflammation *via* the action of pro-inflammatory cytokines (Figure 1).

The latest genomic and epidemiological studies have shown that inflammation and immune responses in the brain are key factors in the pathogenesis and progression of AD (Vanitallie, 2017). Therefore, using anti-inflammatory factors, such as those in the ECM, to relieve pro-inflammatory responses is a viable strategy for the treatment of AD.

## The ECM Protects Neurons From Neurotoxic Factors in AD

Fibrillar Aβ plays a central role in neurotoxicity in AD brains (Mucke and Selkoe, 2012); it was shown to induce oxidative damage and destroy the cytosolic Ca^2+^ homeostasis of hippocampal neurons (Resende et al., 2007). Studies have shown that Aβ promotes an increase in the concentration of Ca^2+^ in the cytoplasm of neurons (Kuchibhotla et al., 2008), which increases the excitability of neurons and causes an increase in the frequency of action potentials (AP; Scarnati et al., 2020). The increase in neuronal AP frequency may be related to the shortened refractory period and lower threshold potential (Tamagnini et al., 2015), which is a common cause of neuronal death in most degenerative diseases. Despite being the main component of senile plaques in AD, Aβ protein does not show neurotoxicity to CSPG-containing neurons. However, when CSPG is removed with ChABC, Aβ1–42 becomes neurotoxic to neurons (Miyata et al., 2007), suggesting that the neuroprotective properties of PNNs could be harnessed as an effective treatment in AD. Furthermore, DSPGs in the ECM prevent the neurotoxic factors within and around senile plaques from interacting with the nearby neurons or astrocytes, which restricts the further spread of neurotoxic effects. Accordingly, neurons wrapped in DSPG-rich ECM are not susceptible to Aβ’s toxic effects (Díaz-Nido et al., 2002). Therefore, an increase in DSPGs may play a key role in reducing amyloid neurotoxicity. The “two-sidedness” of the effects of CSPG and DSPG in AD allows researchers to selectively eliminate or enhance the expression of CSPG or DSPG.

## Conclusion

The pathogenesis of AD is complex. In this article, we found that the same ECM may participate in the initiation and development of AD through a variety of ways, while different ECMs may participate in the pathogenesis of AD *via* the same pathway. This review thus provides a broader basis for further understanding AD pathogenesis.

In summary, different components of the ECM have different roles in the neuropathology of AD, and regulating the expression of individual components is an important step to stabilize or improve the course of the disease. For example, as shown in Figure 2, HA, CSPG, TNR, and other components make up PNNs, which together maintain the stability of the extracellular environment. There may be potential interactions between the different components of the ECM, so that the ECM may participate in a variety of ways. Although the ECM component has various functions, it is also specific in different pathologies of AD. Therefore, this special ECM component will be a potential target and biomarker for the development and treatment of AD. This review describes the possible relevant role of some components of the ECM in AD, but the molecular mechanisms underlying the pathogenesis of AD remain unclear. Therefore, further elucidating the contributions of other ECM components in the pathogenesis of AD will be of great significance for its treatment and that of other AD-related neurodegenerative diseases.

## Author Contributions

QY and YS made substantial contributions to the conception and design of the review and gave final approval of the version to be published. SX, MJ, LY, XL, and ZB participated in writing the particular sections of the manuscript and approved the final version. QY and YS prepared figures. All authors contributed to the article and approved the submitted version.

## Conflict of Interest

The authors declare that the research was conducted in the absence of any commercial or financial relationships that could be construed as a potential conflict of interest.

## Publisher’s Note

All claims expressed in this article are solely those of the authors and do not necessarily represent those of their affiliated organizations, or those of the publisher, the editors and the reviewers. Any product that may be evaluated in this article, or claim that may be made by its manufacturer, is not guaranteed or endorsed by the publisher.
